# Clinical Response to Vedolizumab in Ulcerative Colitis Patients Is Associated with Changes in Integrin Expression Profiles

**DOI:** 10.3389/fimmu.2017.00764

**Published:** 2017-07-03

**Authors:** Friederike Fuchs, Daniela Schillinger, Raja Atreya, Simon Hirschmann, Sarah Fischer, Clemens Neufert, Imke Atreya, Markus F. Neurath, Sebastian Zundler

**Affiliations:** ^1^Kussmaul Campus for Medical Research and Translational Research Center, Department of Medicine 1, Friedrich-Alexander-Universität Erlangen-Nürnberg (FAU), Erlangen, Germany

**Keywords:** inflammatory bowel diseases, ulcerative colitis, T cells, vedolizumab, integrins

## Abstract

**Background:**

Despite large clinical success, deeper insights into the immunological effects of vedolizumab therapy for inflammatory bowel diseases are scarce. In particular, the reasons for differential clinical response in individual patients, the precise impact on the equilibrium of integrin-expressing T cell subsets, and possible associations between these issues are not clear.

**Methods:**

Blood samples from patients receiving clinical vedolizumab therapy were sequentially collected and analyzed for expression of integrins and chemokine receptors on T cells. Moreover, clinical and laboratory data from the patients were collected, and changes between homing marker expression and clinical parameters were analyzed for possible correlations.

**Results:**

While no significant correlation of changes in integrin expression and changes in outcome parameters were identified in Crohn’s disease (CD), increasing α4β7 levels in ulcerative colitis (UC) seemed to be associated with favorable clinical development, whereas increasing α4β1 and αEβ7 correlated with negative changes in outcome parameters. Changes in α4β1 integrin expression after 6 weeks were significantly different in responders and non-responders to vedolizumab therapy as assessed after 16 weeks with a cutoff of +4.2% yielding 100% sensitivity and 100% specificity in receiver-operator-characteristic analysis.

**Discussion:**

Our data show that clinical response to vedolizumab therapy in UC but not in CD is associated with specific changes in integrin expression profiles opening novel avenues for mechanistic research and possibly prediction of response to therapy.

## Introduction

Inflammatory bowel diseases (IBD) with the main entities of Crohn’s disease (CD) and ulcerative colitis (UC) arise from a complex pathogenesis that crucially involves pro-inflammatory T cells (1–3). Most available therapies including the monoclonal anti-α4β7 integrin antibody vedolizumab prominently target these T cells and mediate their beneficial effect on chronic intestinal inflammation by controlling numbers and function of intestinal T cells (4).

While this in some cases includes the promotion of T cell apoptosis (5) or inhibition of pro-inflammatory differentiation (6), vedolizumab is thought to reduce replenishment of intestinal T cells by impeding α4β7 integrin-dependent gut homing (7, 8). Gut homing is a multistep-process facilitating the access of effector and effector memory T cells that have been primed in the gut-associated lymphoid tissue in the presence of retinoid acid to the intestinal lamina propria (9, 10). This process crucially depends on tight adhesion of T cell-expressed α4β7 integrin to endothelial mucosal vascular addressin cell adhesion molecule (MAdCAM)-1 and, consistently, recent *in vitro* and *in vivo* data have shown that vedolizumab mechanistically blocks adhesion of α4β7-expressing T lymphocytes to endothelial MAdCAM-1 (11–13). This is thought to lead to reduced infiltration of pro-inflammatory T cells to the gut with subsequent decrease in inflammation (14).

While vedolizumab has developed to a new mainstay in the therapy of IBD and is successfully used throughout the world (15–17), deeper insights into the immunological effects of α4β7 blockade are still scarce. In particular, the reasons why some patients show no clinical response are still unclear and the factors influencing mucosal healing in vedolizumab-treated patients are largely unknown. Moreover, several pieces of evidence suggest that the efficacy in CD and UC might be different (7, 8, 18), and only partial explanations for these observations are available.

In the present study, we reasoned that different degrees of clinical response to vedolizumab therapy might reflect in different changes in the expression of α4β7 integrin and related T cell surface markers. Accordingly, we sequentially analyzed integrin expression profiles in CD and UC patients receiving clinical vedolizumab therapy and show that several clinical features of disease activity are correlated with specific changes in integrin expression in UC but not CD, which might even serve for prediction of therapeutic response.

## Materials and Methods

### IBD Patients

Patients with established diagnosis of UC (*n* = 17) and CD (*n* = 19) were treated with vedolizumab according to established clinical protocols (7, 8) at the Department of Medicine 1 of the University Hospital Erlangen. Peripheral blood samples were sequentially collected before each treatment from treatment one (T1) up to treatment six to eight (T2–T6/8) with T1–T3 administered at weeks 0, 2, and 6 and T4–T8 administered in intervals of between 4 and 8 weeks depending on clinical response (Figure S1A in Supplementary Material). Table 1 summarizes the patients’ clinical data. Gut samples from control and IBD patients came from surgical specimens or biopsies obtained during routine colonoscopy.

**Table 1:** Patient characteristics.

		Crohn’s disease	Ulcerative colitis
Number		19	17
Age (Ø)		41.7 (20–64)	44.7 (24–68)
Female (%)		68.4	47.1
Harvey–Bradshaw index (Ø)		8.5 (2–21)	
Mayo c.s. (Ø)			3.8 (1–6)
Adjunctive therapy (%)	Immunosuppressants	15.8	17.6
	Steroids	26.3	76.5
	Mesalazin	21	70.5
Previously received anti-TNF therapy (%)		100	88.2
Localization (%)		L1: 10.5	Proctitis: 5.9
		L2: 5.3	Proctosigmoiditis: 17.6
		L3: 42.1	Left-sided colitis: 5.9
		L4+: 36.8	Extended colitis: 5.9
		n.d.: 5.3	Pancolitis: 64.7

*TNF, tumor necrosis factor; n.d., not determined*.

All subjects gave written informed consent in accordance with the Declaration of Helsinki. The protocol was approved by the Ethics Committee of the University Hospital Erlangen.

### Flow Cytometry

Using density gradient centrifugation with Pancoll (Pan Biotech), peripheral blood mononuclear cells were isolated and stained with antibodies against CD4 (VioBlue, VIT4; Miltenyi Biotec), CD8 (AF647, SK1; Biolegend), α4 integrin (FITC, MZ18-24A9; Miltenyi Biotec), αE integrin (PE/Cy7, Ber-ACT8; Biolegend), β1 integrin (AF647, TS2/16; Biolegend), β7 integrin (PerCP/Cy5.5, FIB27; Biolegend), CCR2 (BV605, K036C2; Biolegend), or CCR6 (PE/Cy7, Ber-ACT8; Biolegend) and fixed with the FoxP3/Transcription Factor Staining Buffer Set (eBioscience). Flow cytometric analyses (Figures S1B,C in Supplementary Material) were performed on an LSR Fortessa instrument (BD).

### Immunohistochemistry

For fixation, cryosections of gut samples were incubated with 4% paraformaldehyde. Subsequently, avidin/biotin blocking reagent (Vector Laboratories), protein-blocking reagent (Roth), and goat serum were used for blockade of unspecific binding sites. Slides were incubated with primary antibodies specific for E-cadherin (36/E; BD) and αE integrin [EPR4166(2); Abcam] with subsequent treatment with biotin-conjugated goat anti-mouse antibody (Vectorlabs) and a streptavidin-Dylight 488 conjugate (Biolegend) or a Cy3-labeled goat anti-rabbit antibody (Merck), respectively. After counterstaining of cell nuclei with Hoechst dye (molecular probes), confocal microscopy (LSM SP8) was used for analysis.

### Clinical Parameters

Clinical data documented by the attending physician before treatment initiation or on the occasion of vedolizumab treatments of the analyzed patient cohort were retrospectively collected from the electronic patient files. Particularly, these data included weight (in kilograms), abdominal pain (patient-reported numeric rating scale intensity ranging from 0 to 10), stool frequency (stools per day) and consistency (1—solid, 2—soft, 3—pasty, 4—liquid), presence of blood in the stool, laboratory parameters [C-reactive protein (CRP), hemoglobin], and well-established disease activity indices [Harvey–Bradshaw index (HBI) for CD (19) and Mayo clinical subscore (MCS) for UC (20)].

### Statistics

To correlate changes in integrin expression with clinical parameters, flow cytometric and clinical data from T2 to T8 were analyzed in comparison to the baseline value obtained before T1. Absolute differences compared with T1 (e.g., Δ HBI vs. T1), or relative differences compared with T1 expressed as % of the baseline value (e.g., % α4β1 expression compared with T1) were calculated. Accordingly computed values for integrin and chemokine receptor expression were correlated with the listed clinical parameters in GraphPad Prism, and Pearson’s *r* was calculated. Where reasonable, changes in categorial variables were grouped to “decrease,” “no change,” and “increase,” and corresponding integrin expression changes were compared with one-way ANOVA and Newman–Keuls *post hoc* or Student’s *t*-test.

For the analysis of relation between α4β1 expression changes at T3 and clinical response at T5, UC patients were classified as “responders,” when the MCS had dropped by two or more points from T1 to T5 and as “non-responders,” when the MCS had increased, remained the same, or dropped by not more than one point. Integrin expression changes in these groups were compared by Student’s *t*-test, and a receiver-operator characteristic (ROC) was compiled.

Levels of significance are indicated by asterisks (*p < 0.05, **p < 0.01, ***p < 0.001).

## Results

### Significant Correlation of Changes in Integrin and Chemokine Receptor Expression under Vedolizumab Therapy

We analyzed changes in the expression of integrins and chemokine receptors in a cohort of 19 patients with CD and 17 patients with UC (Table 1).

**Figure 1:**
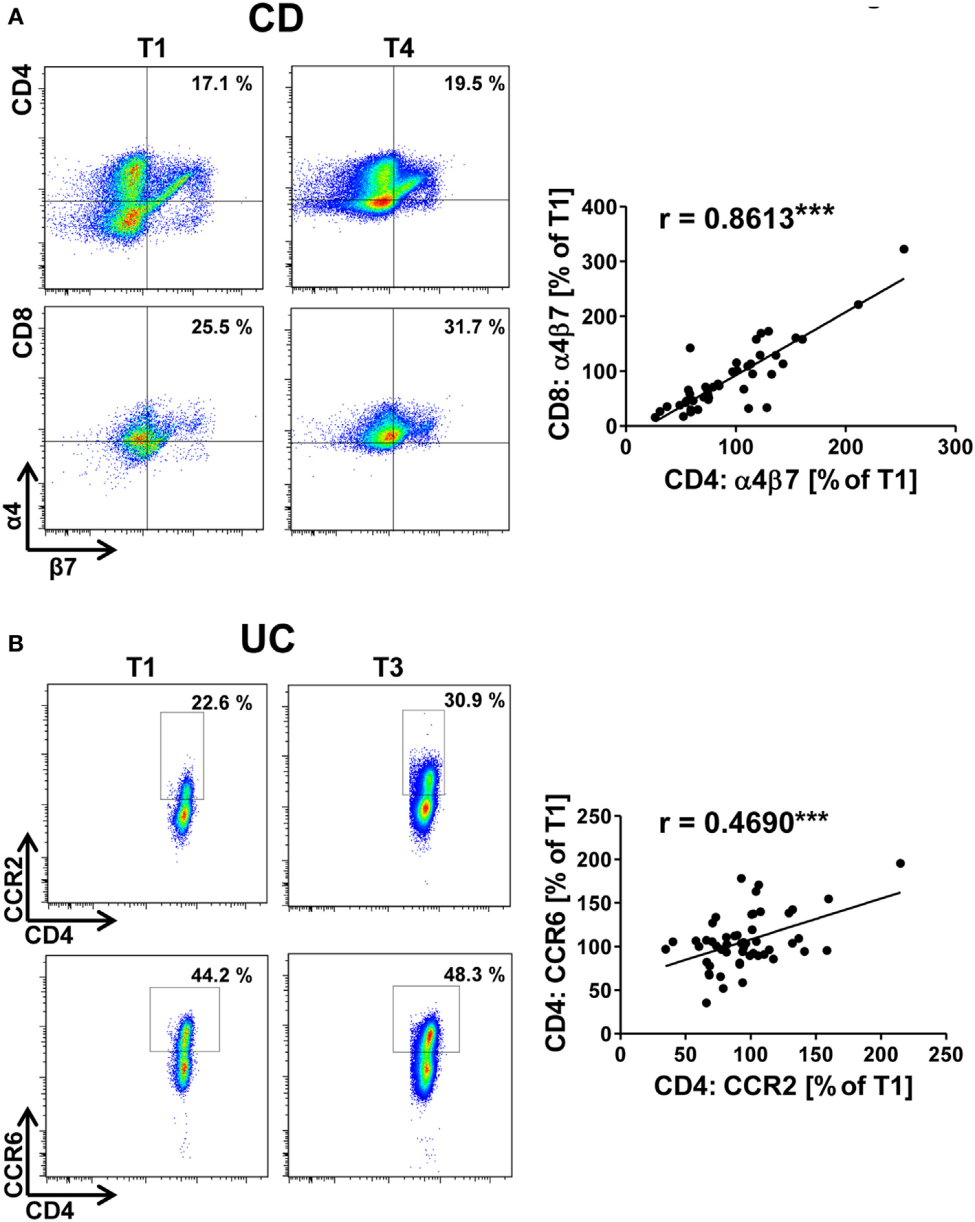
Correlation of dynamic changes in integrin and chemokine receptor expression in patients under vedolizumab treatment. Correlation of changes vs. baseline (T1) observed before treatment 2–8 (T2–8) in flow cytometric α4β7 expression on peripheral CD4^+^ and CD8^+^ T cells from Crohn’s disease (CD) patients **(A)** and of changes in flow cytometric CCR2 and CCR6 expression on peripheral CD4^+^ T cells from ulcerative colitis (UC) patients **(B)** treated with vedolizumab. Left panels: representative plots from one patient showing the percentage of α4^+^β7^+^ among CD4^+^ and CD8^+^ T cells **(A)** and the percentage of CCR2^+^ and CCR6^+^ among CD4^+^ T cells **(B)** at baseline (T1) and before treatment 3 or 4 (T3/T4) as indicated. Right panel: pooled data from 12 **(A)** and 15 patients **(B)** depicting the changes vs. T1 observed before T2 to T8. Pearson’s *r* and significances are indicated.

Since the factors regulating integrin and chemokine receptor expression in different T cell subsets substantially intersect (21, 22), we reasoned that tracking expression of such markers in patients over time should reveal concordant changes between different subsets or different markers. Thus, we started our analyses with according explorations. We found a significant correlation of changes in the α4β7 integrin expression on CD4^+^ with that on CD8^+^ T cells both in CD (Figure 1A) and in UC (Figure S2A in Supplementary Material). A similar finding was made for the correlation of changes in the expression of CCR2 with CCR6 on CD4^+^ T cells (Figure 1B; Figure S2B in Supplementary Material). Moreover, changes in αEβ7 integrin expression on CD4^+^ and CD8^+^ T cells were correlated with each other and an association of changes in α4β1 expression with both CCR2 and CCR6 was found in CD (Figures S2C–E in Supplementary Material and data not shown), confirming that cues regulating integrin expression in T cells have similar impact on the CD4^+^ and the CD8^+^ subset and suggesting that there is considerable overlap in the signals regulating expression of homing markers.

### Changes of α4β7 Integrin Expression Are Related to Clinical Presentation of Vedolizumab-Treated Patients in UC but Not in CD

In addition, we correlated the changes in the expression of integrins and chemokine receptors over the course of vedolizumab therapy to changes in clinical parameters.

**Figure 2:**
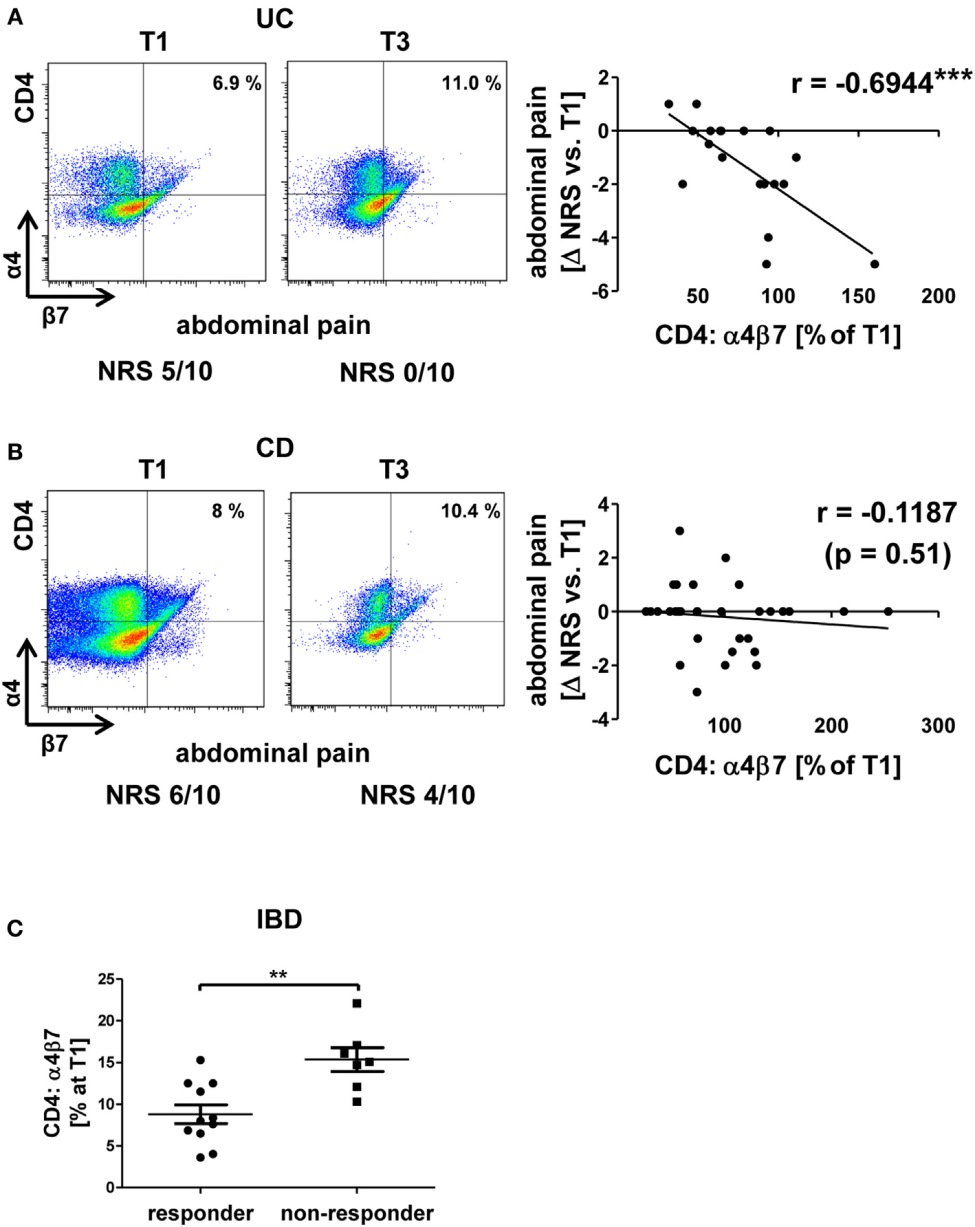
Correlation of dynamic changes in α4β7 integrin expression with clinical parameters in patients under vedolizumab therapy. **(A,B)** Correlation of changes vs. baseline (T1) observed before treatment 2–8 (T2–8) in flow cytometric α4β7 expression on peripheral CD4^+^ T cells with changes in patient-reported abdominal pain in patients with UC **(A)** and CD **(B)**. Left panels: representative plots from one patient showing the expression of α4β7 on CD4^+^ T cells before the mentioned treatments and indicating the corresponding abdominal pain rating below. Right panels: pooled data from 7 **(A)** and 11 patients **(B)** depicting the changes vs. T1 observed at T2–T8. Pearson’s *r* and significances are indicated. IBD, inflammatory bowel diseases; NRS, numerical rating scale; CD, Crohn’s disease; UC, ulcerative colitis. **(C)** Flow cytometric expression of α4β7 integrin at baseline in IBD patients with a clinical response (defined as decrease of at least two points in Mayo clinical subscore or Harvey–Bradshaw index) after 16 weeks. Significance is indicated.

For α4β7 integrin, we found that increasing expression on CD4^+^ T cells from patients with UC during vedolizumab therapy was associated with decreasing abdominal pain reported by the patients as numeric rating scale intensity (Figure 2A). This might reflect successful blockade of α4β7-dependent gut homing leading to an increasing percentage of α4β7-expressing T cells in the peripheral blood and, consistently, to reduced intestinal symptoms.

Unexpectedly, however, no such association could be identified for patients with CD (Figure 2B). This is consistent with the notion that response or non-response to vedolizumab therapy in CD does not go along with specific alterations of α4β7 integrin expression and suggests that differences between the mechanistic impact of vedolizumab therapy in CD and UC exist.

In addition, we wondered whether response or non-response to vedolizumab might be associated with different pretreatment levels of α4β7-expressing CD4^+^ T cells. Surprisingly, it appeared that IBD patients with a clinical response after 16 weeks (defined as a decrease of at least two points in the HBI or MCS) had lower initial frequencies of α4β7-expressing T cells than patients without clinical response (Figure 2C). While this finding requires prospective validation in larger cohorts, it might indicate that low α4β7 expression increases the likelihood that α4β7-dependent homing of disease-relevant T lymphocytes to the gut is completely blocked.

### Dynamic Expression of αEβ7 Integrin on T Cells Is Associated with Clinical Outcome Parameters in UC

**Figure 3:**
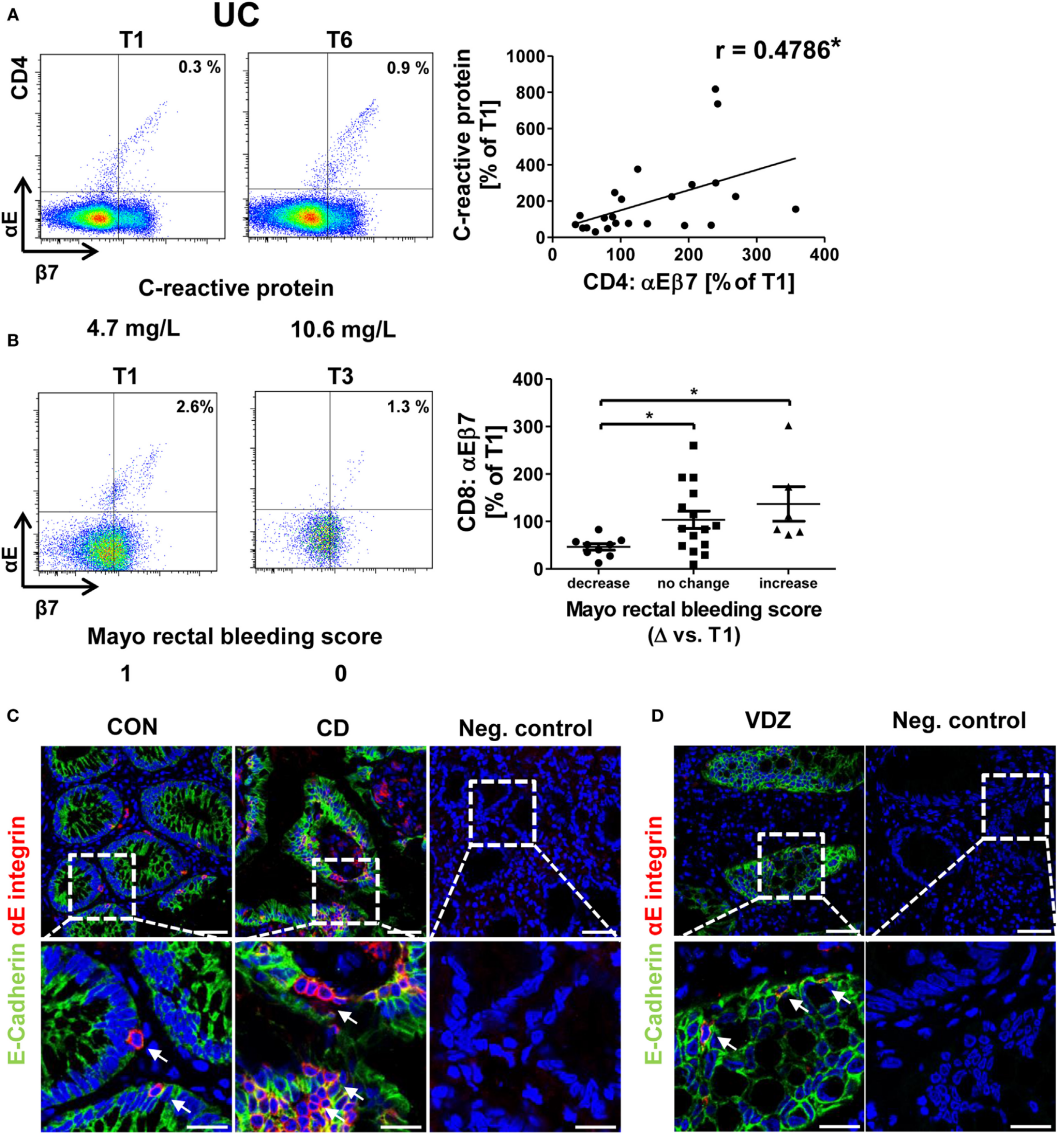
Correlation of dynamic changes in αEβ7 integrin expression with clinical parameters in patients under vedolizumab therapy. **(A)** Correlation of changes vs. baseline (T1) observed before treatment 2–8 (T2–8) in αEβ7 expression on peripheral CD4^+^ UC T cells with changes in C-reactive protein levels. **(B)** Changes in αEβ7 expression on peripheral CD8^+^ UC T cells in patients with decreasing, unchanged, or increasing Mayo rectal bleeding score. Left panels: representative plots from one patient showing the expression of αEβ7 on CD4^+^ or CD8^+^ T cells before the mentioned treatments and indicating the corresponding clinical parameters below. Right panels: pooled data from 9 patients depicting the changes vs. T1 observed at T2–T8. Pearson’s *r* and significances are indicated. **(C,D)** Representative images showing immunohistochemistry of gut cryosections for αEβ7 (red) and epithelial E-cadherin (green) in a non-IBD patient (CON) and a CD patient **(C)** as well as in a patient treated with vedolizumab **(D)**. White arrows indicate αEβ7^+^ cells in contact with E-cadherin^+^ epithelial cells. VDZ, vedolizumab; IBD, inflammatory bowel diseases; CD, Crohn’s disease; UC, ulcerative colitis.

Moreover, an association of rising αEβ7 expression with worse development of clinical parameters was noted in UC: there was a coherence of increases in αEβ7 expression on CD4^+^ T cells with increasing levels of the inflammation marker CRP (Figure 3A) and a trend toward looser stools when αEβ7 expression increased (Figure S3A in Supplementary Material). Such association of rising αEβ7 with poorer clinical presentation was even clearer when analyzing αEβ7 on CD8^+^ T cells. Here, relative αEβ7 expression compared with T1 was significantly increased in patients with mounting scores in the MCS “rectal bleeding score” component compared with patients with declining scores (Figure 3B). This was backed up by a highly significant correlation of increasing αEβ7 on CD8^+^ T cells with looser stool consistency and increasing CRP. Moreover, a strong trend for a positive coherence with increasing abdominal pain was noted (Figure S3B in Supplementary Material). These observations proposed that increasing αEβ7 might have a negative impact on the outcome of vedolizumab therapy in UC. Once again, no similar correlations could be identified in CD (data not shown).

Some of these observations for the correlation of αEβ7 with clinical data suggested a link of αEβ7 with intestinal epithelial barrier integrity, since normal consistency and frequency of bowel movements as well as the absence of blood in the stool require an intact epithelium to allow resorption of nutrients and foods as well as to preserve the integrity of deeper layers of the gut wall.

Accordingly, we performed immunohistochemical stainings for αE integrin and its ligand, the epithelial cell marker E-cadherin. As expected, we could demonstrate αE^+^ cells occurring in close proximity to epithelial cells both in the healthy and inflamed gut (Figure 3C) and, furthermore, also in patients receiving vedolizumab (Figure 3D). Although our sequential measurements confined to the peripheral blood, this indicated that the reason for specific association of dynamic αEβ7 expression changes with clinical development under vedolizumab therapy might be due to an impact of αEβ7-expressing T cells on the intestinal epithelium.

### Changes in α4β1 Expression after 6 Weeks Vedolizumab in UC Are Correlated with Clinical Response after 16 Weeks

**Figure 4:**
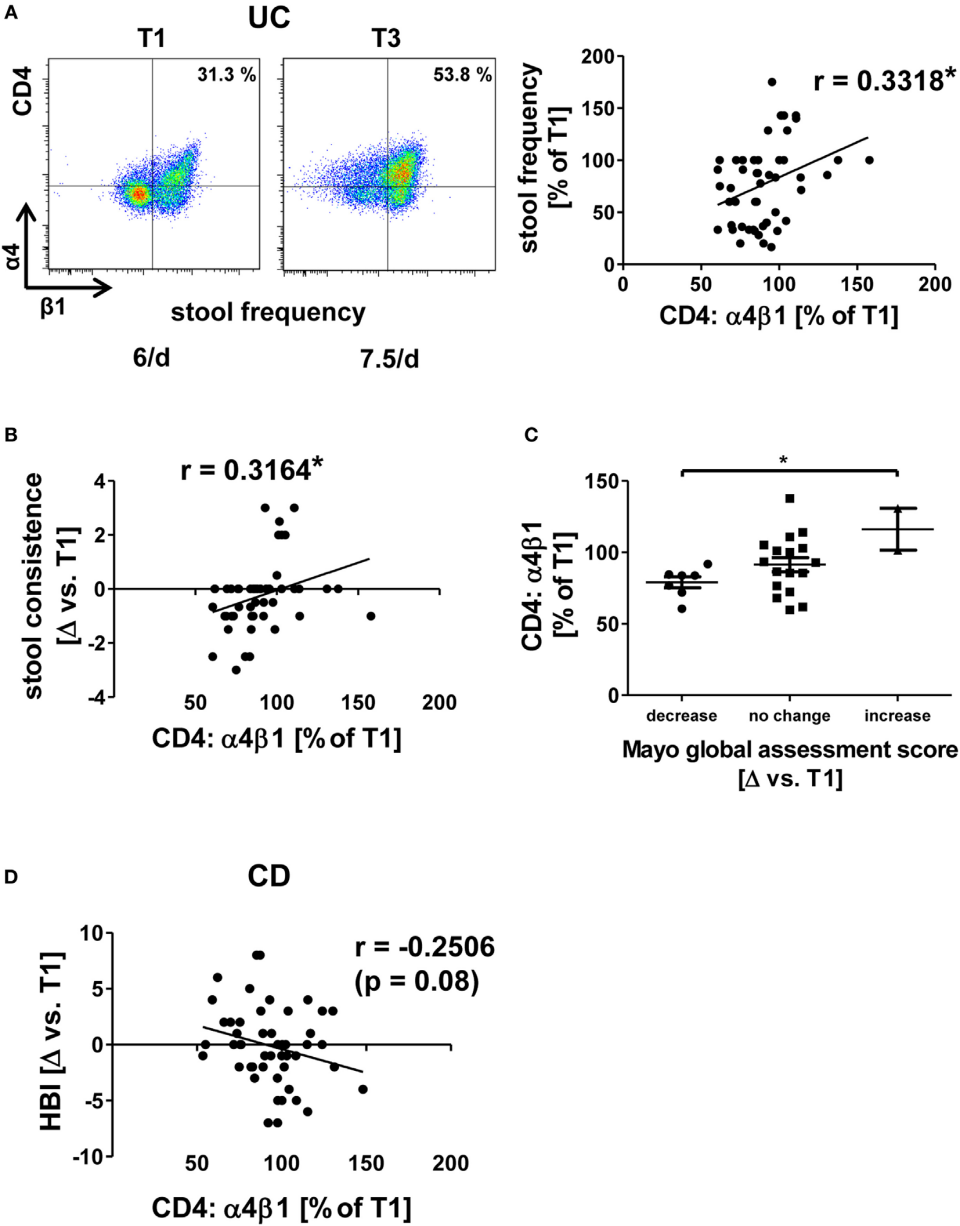
Correlation of dynamic changes in α4β1 integrin expression with clinical parameters in patients under vedolizumab therapy. **(A,B,D)** Correlation of changes vs. baseline (T1) observed before treatment 2–8 (T2–8) in flow cytometric α4β1 expression on peripheral CD4^+^ T cells with changes in stool frequency **(A)** and stool consistence **(B)** in ulcerative colitis (UC) patients and Harvey–Bradshaw index (HBI) in Crohn’s disease (CD) patients **(D)**. Representative plots from one patient **(A)** show the expression of α4β1 on CD4^+^ T cells before the mentioned treatments, and the corresponding stool frequency is indicated below. Graphs **(A,B,D)** show pooled data from 13 to 14 patients depicting the changes vs. T1 observed at T2–T8. Pearson’s *r* and significances are indicated. **(C)** Changes vs. T1 in flow cytometric α4β1 expression on peripheral CD4^+^ T cells observed before T2–T8 in UC patients with decrease, no change, or increase of the Mayo global assessment subscore. Significance is indicated.

A similar pattern of association of dynamic integrin expression with clinical outcome parameters as for αEβ7 integrin was identified for α4β1 integrin in UC since increases in α4β1 expression were correlated with worse development of clinical parameters in vedolizumab-treated patients. Particularly, when α4β1 rose, patients experienced a higher frequency of bowel movements (Figure 4A). Moreover, when patients reported of looser stools compared with T1, they were more likely to have increased levels of α4β1 expression compared to T1, resulting in a significant correlation of these parameters (Figure 4B). This is also consistent with the finding that in patients, in which the partial “physician global assessment score” of the MCS dropped, relative α4β1 expression compared to T1 was lower than in those with increasing physician global assessment scores (Figure 4C).

However, no significant correlation between clinical changes and α4β1 could be identified in CD. Yet, there was a trend (*p* = 0.08) suggesting that decreasing α4β1 expression might be associated with increasing HBI scores in CD patients (Figure 4D). Taken together, dynamic changes in all α4β7-related integrins were significantly related to dynamic changes of clinical outcome parameters in UC, which is compatible with the perception that individual (counter-)regulatory pathways might affect outcome of vedolizumab therapy in UC by mediating the expression of integrins. On the other hand, while the findings for α4β1 integrin rather suggested a differential regulation compared with UC, no such significant correlations were identified in CD supporting the idea that molecular differences in the homing pathways implicated in CD and UC exist.

Like for other drugs, the response to vedolizumab treatment cannot be predicted in single patients so far leading to a significant portion of patients, which are treated without success and have to be assigned to another therapy. In this light, we explored whether any of the above depicted findings might be used to identify an early marker of successful vedolizumab treatment in UC. To this end, we compared integrin expression changes after 6 weeks of vedolizumab treatment (i.e., before T3) with clinical outcome before T5 (i.e., 16.1 ± 0.2 weeks), and patients were classified as “responders” and “non-responders” based on the MCS as described in the Section “Materials and Methods.”

**Figure 5:**
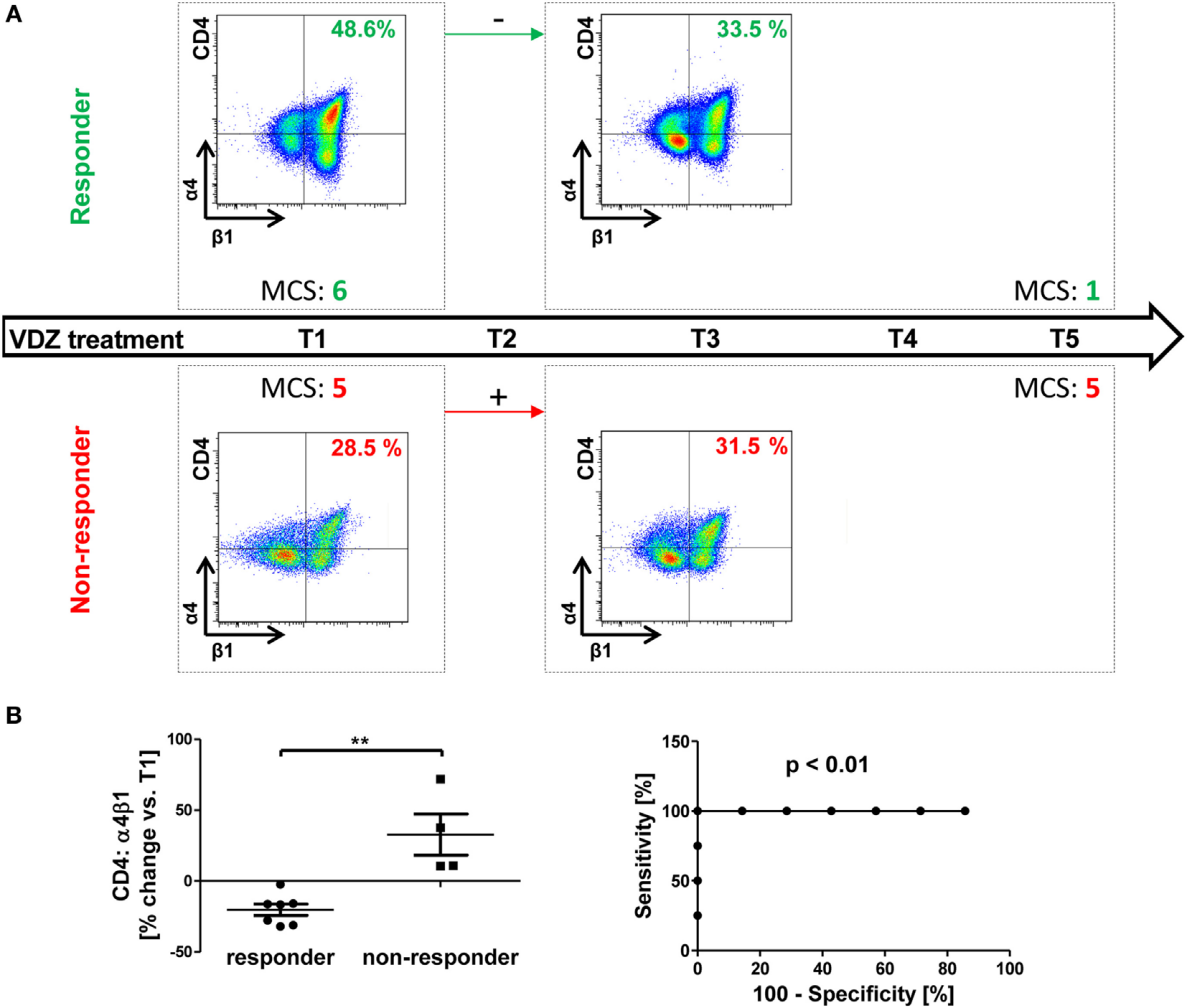
Possible prediction of response to vedolizumab therapy by changes in α4β1 integrin expression in ulcerative colitis (UC). **(A)** Schematic drawing representatively depicting the association of changes in α4β1 expression from baseline (T1) to treatment three (T3) with the response to vedolizumab therapy from T1 to T5 in UC patients. Upper panels: in patients with Mayo clinical subscores (MCS) decreasing by at least two points (responders, green) α4β1 expression on CD4^+^ T cells decreased. Lower panels: in patients without clear decrease in the MCS, α4β1 expression increased. **(B)** Left panel: pooled statistic of the evaluated cohort (*n* = 11). Right panel: receiver-operator-characteristic analysis for prediction of clinical response before T5 by changes in α4β1 observed before T3. Significances are indicated.

Indeed, we found that patients with a clinical response had decreasing α4β1 levels after 6 weeks compared with baseline, while patients without clinical response had increasing levels compared with baseline (Figure 5A).

This was the case in all 11 patients that could be included into this analysis, and the distribution was statistically significant. An ROC analysis showed that a cutoff of +4.2% change in α4β1 integrin from T1 to T3 had 100% sensitivity and 100% specificity for the allocation of patients from our cohort to the responder or non-responder group at T5 (Figure 5B). Of note, no association of initial levels of α4β1-expressing CD4^+^ T cells with response could be observed, and only one of seven responders already fulfilled the respective criterium of at least two points drop in MCS at T3, indicating that changes in α4β1 expression are indeed preceding clinical outcome manifestation.

## Discussion

The approval of vedolizumab for clinical therapy of both UC and CD has substantially increased the therapeutic armamentarium in IBD (7, 8). Meanwhile, efficacy and safety have not only been documented in randomized clinical trials but also in real-world settings (15–17). This has been accompanied by mechanistic investigations elucidating *in vivo* effects of vedolizumab on T cell homing (13, 23). However, a number of questions regarding the immunological effects of vedolizumab remain. For instance, it remains elusive why vedolizumab lacks effect in a portion of patients and why this portion seems to be larger in CD compared with UC (7, 8, 18). Moreover, this also includes questions addressing the immunological sequelae of α4β7-dependent homing disruption, e.g., regarding the expression and functionality of other homing molecules in view of their effects on the equilibrium of peripheral blood and intestinal T cell populations (14).

Our present study was conducted with the aim to bring some light into these uncertainties and, therefore, we systematically analyzed associations of changes in α4β7 and related integrins (24) as well as in chemokine receptors with changes in clinical parameters over the course of vedolizumab therapy. For the first time, our data show that several parameters of patient-reported and physician-documented response to vedolizumab treatment are associated with specific changes in the expression of integrins but not chemokine receptors in UC providing new insights into the mechanisms of vedolizumab therapy and fueling hopes for their use in prediction of response to therapy.

While several significant correlations between integrin expression changes and clinical parameter changes were identified in UC, none could be identified in CD. On a molecular level, this further substantiates the empirical clinical observation that differences in the efficacy of vedolizumab treatment seem to exist between UC and CD (18). The only correlation that was approaching significance was that of changes in α4β1 expression with changes in HBI score, suggesting that decrease of the former might go along with increase of the latter parameter. This is in line with earlier observations in an *in vivo* mouse model showing that compensatory homing *via* the α4β1/vascular cell adhesion molecule (VCAM)-1 pathway might bypass α4β7 blockade in CD (23) and matching to rodent data that propose considerable redundancy in different homing pathways (25, 26) and VCAM-1-dependent homing as an important pathway in CD-like experimental colitis (27). Accordingly, decreasing α4β1^+^ CD4 T cells in the peripheral blood might reflect increased gut homing of such cells triggering increased intestinal inflammation.

In UC, increase in α4β7 seemed to be associated with favorable clinical development, while increase of α4β1 or αEβ7 expression were correlated with worsening of several clinical parameters. The specificity of these findings for integrins was supported by the fact that changes in CCR2 and CCR6 expression, which are primarily unrelated to α4β7, did not correlate with any of the parameters analyzed.

While these observations are undoubtedly interesting, they are raising new questions regarding the underlying mechanisms. A possible explanation of our findings could be that patients in which α4β7 blockade by vedolizumab sufficiently works have both more peripheral blood T cells expressing α4β7 and amelioration of clinical symptoms due to preclusion of α4β7^+^ T cells from the gut tissue. On the other hand, upregulation of α4β1 and αEβ7 might be a sign of upregulation of rescue pathways, which has also been proposed to be responsible for extraintestinal side effects observed under vedolizumab therapy (28). In particular, cells might upregulate the expression of alternative integrins in an attempt to ensure access to or positioning in the lamina propria *via* alternative pathways beyond the blocked α4β7–MAdCAM-1 axis, which could subsequently lead to severer or maintained inflammation. However, especially since the coherence of α4β1 expression changes with changes in clinical parameters seems to be different in CD und UC, this remains speculative and underscores that additional translational research is necessary to better understand the alterations in integrin-expressing cell subsets at the interface of the peripheral blood and the intestine. Yet, in light of the above remarks, such differences between CD and UC must not be surprising but should be interpreted as another cue illustrating differences in therapeutic interference with homing in CD and UC. It has also to be taken into account that our cohort mainly consisted of patients previously exposed to anti-tumor necrosis factor (TNF)-α antibodies, and results might not show such a difference in anti-TNF naïve CD and UC collectives.

No comparable data for the use of anti-adhesion antibodies have been reported so far. However, one study sequentially assessed the peripheral blood of IBD patients under therapy with the anti-TNF-α antibody infliximab for expression of regulatory T cell (Treg) markers. The authors showed that infliximab responders and non-responders had differential development in peripheral Treg profiles (29). Thus, although infliximab is believed to mediate its effect predominantly by inhibition of increased TNF-α signaling in the lamina propria, associated changes could be noted in the blood. Since vedolizumab blocks α4β7 integrin on T cells in the peripheral blood, such analyses even assess the changes of immunological markers at the point of action vedolizumab.

The findings reported for αEβ7 match with a recent report from our group suggesting that a subset of αEβ7^+^ T cells does not express α4β7 (22), and αEβ7^+^ cells might accumulate in the gut *via* additional or alternative pathways. Of note, αEβ7 itself has been proposed to mediate gut homing independently of α4β7 *via* a so far unknown ligand (30). Moreover, it has to be mentioned that additional αEβ7^+^ T cells have been shown to be induced in the gut in response to epithelium-released transforming growth factor-β (31), and the only known ligand for αEβ7 is E-cadherin expressed on the intestinal epithelium (32–34). Thus, it seems possible that cells deprived of α4β7 compensatorily upregulate αEβ7 on their surface in search of another homing pathway to reach the gut or—more general—in search of possibilities to ensure positioning in the lamina propria (whether by homing or by epithelial retention). As we show, many αEβ7-bearing cells can be found in close contact with E-cadherin-expressing epithelium. Thus, it is very likely that αEβ7^+^ T cells communicate with the epithelium. Independent reports have recently shown that αEβ7 is enriched in pro-inflammatory T cell subsets (22, 35) and that αEβ7^+^ T cells express higher levels of granzyme A than αEβ7^−^ T cells (36). *In vivo*, this might result in deleterious effects of such αEβ7-expressing on epithelial cells, which is supported by some of our data showing correlations of changes in αEβ7 integrin with changes in clinical parameters that are indicative of intestinal epithelial barrier function.

Taken together, these correlation analyses indicate so far unknown associations between clinical and immunological parameters, while the exact mutual dependencies need to be clarified in further research. Though, from a clinical perspective the questions whether such associations or initial expression levels might be exploited for monitoring or even prediction of therapeutic response to vedolizumab obtrudes and, thus, we performed respective analyses as detailed above. Unexpectedly, we observed that IBD patients with a clinical response after 16 weeks had lower initial levels of α4β7-expressing CD4^+^ T cells than non-responders. This is intriguing since one could have assumed that higher α4β7 expression is a sign of higher importance of α4β7-dependent homing, and it might thus be more promising to block α4β7 in patients with higher expression. Yet, the explanation for our finding could be that even low numbers of α4β7-expressing T cells are crucial for disease pathogenesis and low initial expression might raise the odds of completely preventing these T lymphocytes from homing to the gut. It will be an important task of future studies to prospectively validate this preliminary observation. Moreover and most interestingly, we also found a surprisingly clear association of changes in α4β1 expression on CD4^+^ T cells after 6 weeks with clinical response after 16 weeks. While these pilot data—like the whole study—are limited by the rather small patient number and retrospective collection of clinical data, thus requiring confirmation in larger multicenter studies, it is nevertheless an observation that disserves further investigation and raises hopes that 2 months of ineffective treatment could be saved in some patients by measurement of the α4β1 expression profile at baseline and after 6 weeks of treatment. Although this would not be a prediction marker that can be assessed before beginning therapy like it was conceptually shown for membrane-bound TNF-α receptor in therapy with anti-TNF-α antibodies (37) or intestinal αE expression in therapy with the experimental anti-β7 integrin antibody etrolizumab (38), it could yet accelerate the assessment of individual response to vedolizumab.

In conclusion, our results suggest that individual response to vedolizumab treatment in UC might be reflected by specific changes in integrin profiles in the peripheral blood. Further studies are required to confirm the translational potential of these observations for the prediction of response to therapy.

## Ethics Statement

All subjects gave written informed consent in accordance with the Declaration of Helsinki. The protocol was approved by the Ethics Committee of the University Hospital Erlangen.

## Author Contributions

FF, DS, and SZ performed the experiments; RA, SH, SF, CN, IA, MN, and SZ provided clinical samples, protocols, reagents, or designed experiments; FF, DS, CN, IA, MN, and SZ analyzed and interpreted the data; SZ drafted the manuscript; all authors critically revised the manuscript for important intellectual content.

## Conflict of Interest Statement

MN has served as an advisor for Pentax, Giuliani, MSD, Abbvie, Janssen, Takeda, and Boehringer. SZ and MN received research support from Takeda and Hoffmann-La Roche. All other authors declare no conflict of interest.

## Supplementary Material

Figure S1 Study outline. **(A)** Schematic sketch of the study design. Patients treated with vedolizumab were followed up from treatment one to six to eight, and blood samples were sequentially collected before each treatment for subsequent flow cytometric analysis of integrin and chemokine receptor expression on CD4^+^ and CD8^+^ T cells. Clinical and laboratory data from the respective patients were retrospectively collected. Changes of clinical and flow cytometric parameters were correlated. **(B,C)** Gating strategy for the measurement of integrin and chemokine receptor expression on T cells. After exclusion of doublets and gating on lymphocytes in the forward/sideward-scatter, CD4^+^ **(B)** or CD4^+^ and CD8^+^ T cells were selected, and the expression of α4^+^β1^high^, CCR2^+^, and CCR6^+^ **(A)** or α4^+^β7^+^ and αE^+^β7^+^ cells **(B)** were quantified, respectively.

Figure S2 Correlation of dynamic changes in integrin and chemokine receptor expression in patients under vedolizumab therapy. Correlation of changes in flow cytometric α4β7 expression on peripheral CD4^+^ and CD8^+^ T cells from ulcerative colitis (UC) patients **(A)**, of changes in flow cytometric CCR2 and CCR6 expression on peripheral CD4^+^ T cells **(B)**, of changes in flow cytometric αEβ7 expression on peripheral CD4^+^ and CD8^+^ T cells **(C)**, and of changes in flow cytometric α4β1 expression with changes in CCR2 **(D)** and CCR6 **(E)** expression on peripheral CD4^+^ T cells in Crohn’s disease (CD) patients treated with vedolizumab. **(C)** Contains representative plots showing the percentage of αE^+^β7^+^ among CD4^+^ and CD8^+^ T cells before the indicated treatments. Panels include pooled data from 12 to 18 patients. Pearson’s *r* and significances are indicated.

Figure S3 Correlation of dynamic changes in integrin expression with clinical parameters in ulcerative colitis (UC) patients under vedolizumab treatment. Correlation of changes in flow cytometric expression of αEβ7 on CD4^+^ T cells **(A)** and CD8^+^ T cells **(B)** with changes in the indicated clinical parameters. Pearson’s *r* and significances are indicated. Panels include data from 9 to 15 patients.

Table S1 Correlation of changes in integrin expression with changes in clinical parameters in ulcerative colitis patients. Pearson’s *r* values for the correlation of changes in expression of the different integrins (in lines) with the changes of a representative panel of clinical parameters (in columns) are noted. Dark green color indicates significant correlations matching with the overall picture mentioned in the text. Light green indicates correlations not reaching significance matching with the overall picture mentioned in the text. Pale green indicates correlations not further supporting the overall picture mentioned in the text. Here, *p* values are additionally indicated to show that these correlations were not essential for overall interpretation.
