# Supplementary figures and images for: Clinical Response to Vedolizumab in Ulcerative Colitis Patients Is Associated with Changes in Integrin Expression Profiles
Source: Front Immunol. 2017 Jul 3;8:764. doi: 10.3389/fimmu.2017.00764 (PMC5495081; doi:10.3389/fimmu.2017.00764)

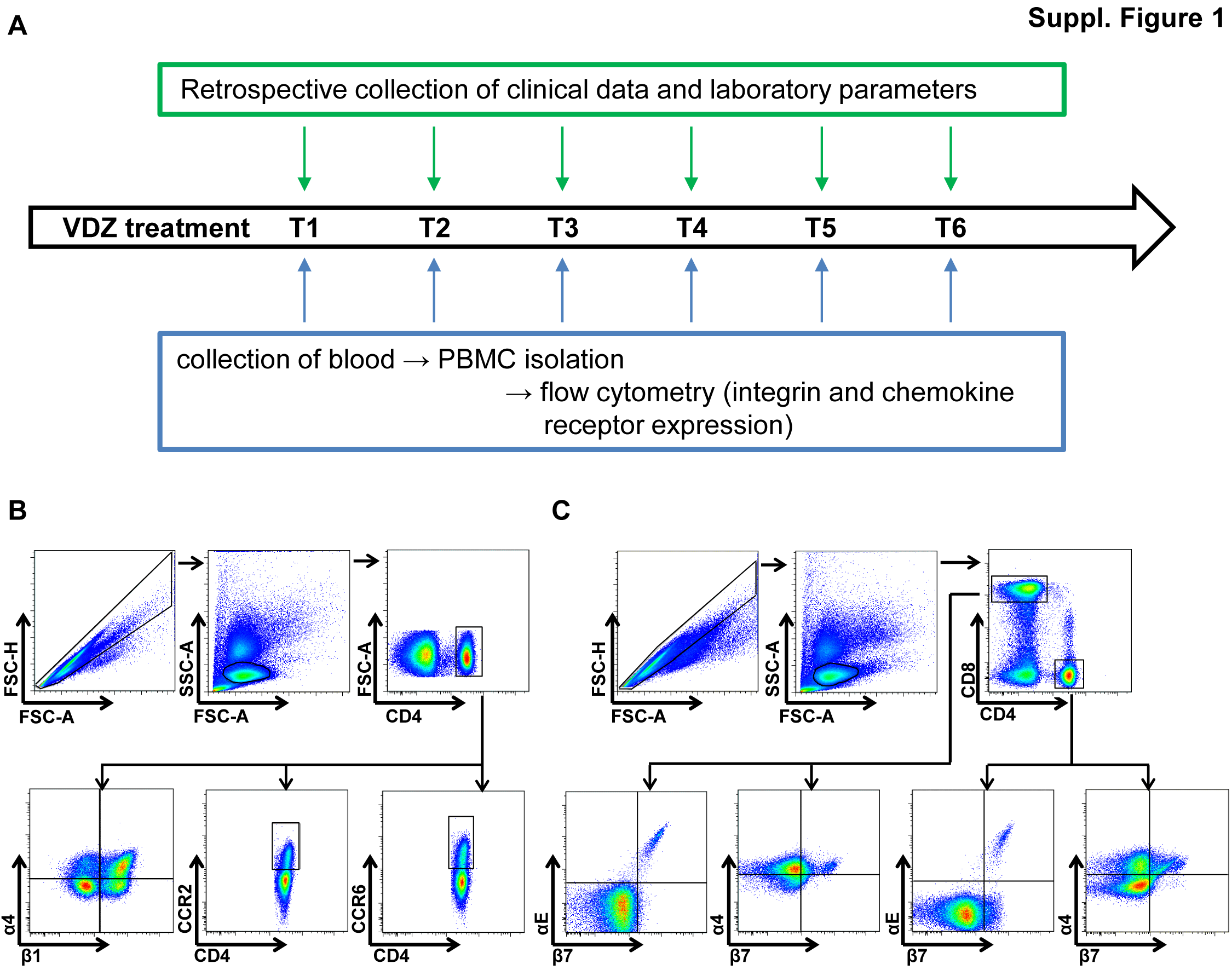

Supplement: Figure S1 — Study outline. (A) Schematic sketch of the study design. Patients treated with vedolizumab were followed up from treatment one to six to eight, and blood samples were sequentially collected before each treatment for subsequent flow cytometric analysis of integrin and chemokine receptor expression on CD4+ and CD8+ T cells. Clinical and laboratory data from the respective patients were retrospectively collected. Changes of clinical and flow cytometric parameters were correlated. (B,C) Gating strategy for the measurement of integrin and chemokine receptor expression on T cells. After exclusion of doublets and gating on lymphocytes in the forward/sideward-scatter, CD4+ (B) or CD4+ and CD8+ T cells were selected, and the expression of α4+β1high, CCR2+, and CCR6+ (A) or α4+β7+ and αE+β7+ cells (B) were quantified, respectively. [file Image_1.tif]

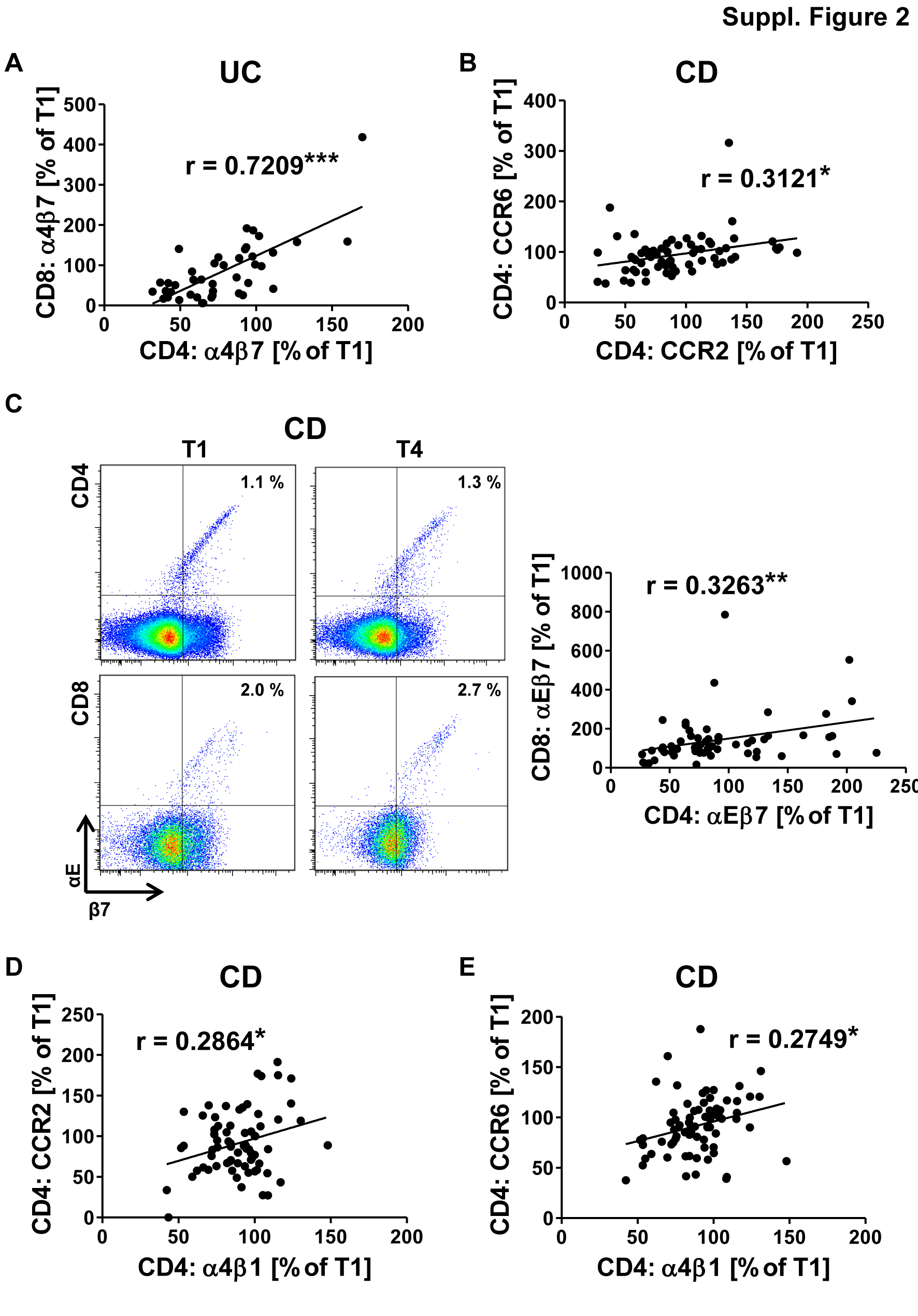

Supplement: Figure S2 — Correlation of dynamic changes in integrin and chemokine receptor expression in patients under vedolizumab therapy. Correlation of changes in flow cytometric α4β7 expression on peripheral CD4+ and CD8+ T cells from ulcerative colitis (UC) patients (A), of changes in flow cytometric CCR2 and CCR6 expression on peripheral CD4+ T cells (B), of changes in flow cytometric αEβ7 expression on peripheral CD4+ and CD8+ T cells (C), and of changes in flow cytometric α4β1 expression with changes in CCR2 (D) and CCR6 (E) expression on peripheral CD4+ T cells in Crohn’s disease (CD) patients treated with vedolizumab. (C) Contains representative plots showing the percentage of αE+β7+ among CD4+ and CD8+ T cells before the indicated treatments. Panels include pooled data from 12 to 18 patients. Pearson’s r and significances are indicated. [file Image_2.tif]

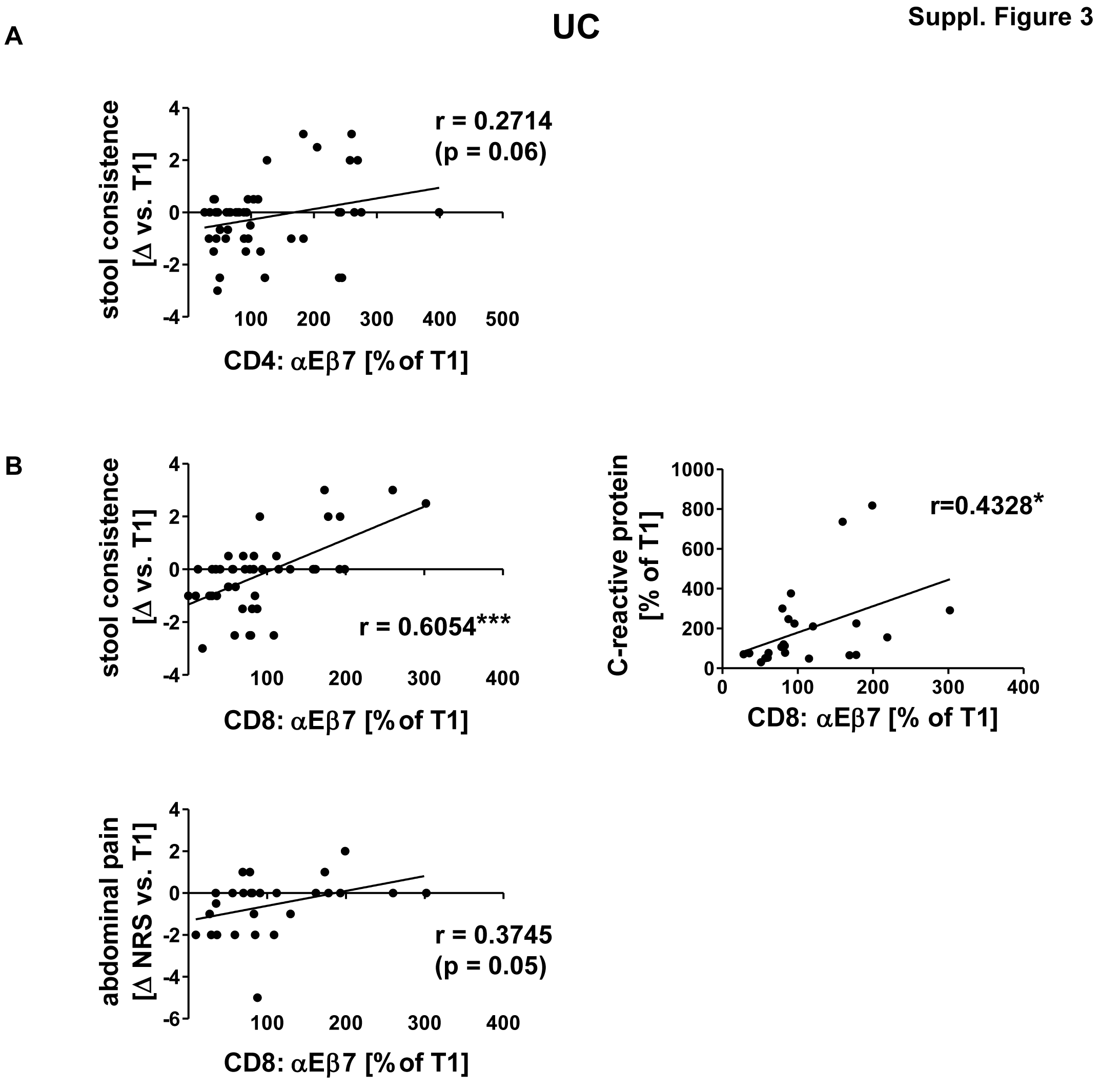

Supplement: Figure S3 — Correlation of dynamic changes in integrin expression with clinical parameters in ulcerative colitis (UC) patients under vedolizumab treatment. Correlation of changes in flow cytometric expression of αEβ7 on CD4+ T cells (A) and CD8+ T cells (B) with changes in the indicated clinical parameters. Pearson’s r and significances are indicated. Panels include data from 9 to 15 patients. [file Image_3.tif]
