# Supplementary material for: Clinical Response to Vedolizumab in Ulcerative Colitis Patients Is Associated with Changes in Integrin Expression Profiles
Source: Front Immunol. 2017 Jul 3;8:764. doi: 10.3389/fimmu.2017.00764 (PMC5495081; doi:10.3389/fimmu.2017.00764)
Supplement: Table S1 — Correlation of changes in integrin expression with changes in clinical parameters in ulcerative colitis patients. Pearson’s r values for the correlation of changes in expression of the different integrins (in lines) with the changes of a representative panel of clinical parameters (in columns) are noted. Dark green color indicates significant correlations matching with the overall picture mentioned in the text. Light green indicates correlations not reaching significance matching with the overall picture mentioned in the text. Pale green indicates correlations not further supporting the overall picture mentioned in the text. Here, p values are additionally indicated to show that these correlations were not essential for overall interpretation. [file Table_1.PDF]

Supplementary Table 1

	abdominal pain	stool frequency	stool consistence	Mayo clinical score	Mayo rectal bleeding subscore	C-reactive protein
**CD4: α4β1**	0.10	0.33*	0.32*	0.15	0.24	-0,14 (p = 0.50)
**CD4: α4β7**	-0.69***	-0.03	0.10 (p = 0.60)	0,10 (p = 0.60)	0.08 (p=0.75)	0,13 (p = 0.74)
**CD4: αEβ7**	0.24	0.05	0.27	0.20	0,33	0,48*
**CD8: αEβ7**	0.37	0.19	0.61***	0,38**	0,47**	0,43*
